# What recent primary studies tell us about ovarian teratomas in children: a scoping review

**DOI:** 10.1007/s10555-020-09844-3

**Published:** 2020-01-31

**Authors:** Justyna Łuczak, Maciej Bagłaj, Piotr Dryjański

**Affiliations:** Pediatric Surgery and Urology Department, Wroclaw Medical University, Wroclaw, Poland

**Keywords:** Ovarian neoplasms, Teratoma, Ovary, Child, Review, Scoping study

## Abstract

Our knowledge of ovarian teratomas in children is still far from complete, and much remains to be discovered. Here, we conduct a scoping review of the primary research related to ovarian teratomas in pediatric age. To our knowledge, there is no published synthesis of the literature surrounding ovarian teratomas in children using scoping review methodology. We identified 24 studies from 11 countries; 18 studies were retrospective, 3 were prospective, and 3 were experimental. There were 6 studies concerning mature teratomas, 5 concerning immature teratomas, and 13 that included both tumor types. Overall, 9 out of all the studies concerned more than 50 patients. We revealed 7 major branches of research within the topic of ovarian teratoma in pediatric population: recurrence rate/relapse and follow-up strategy, malignant potential, prognostic factors, use of sparing surgery, differences between the use of laparoscopy and laparotomy, use of chemotherapy, and additional examinations to test the character of the lesion (immature *vs*. mature). This scoping review has revealed a number of knowledge gaps in the evidence base for pediatric ovarian teratomas. Overall, this topic has not been extensively explored, and more research dedicated exclusively to this tumor and patient population is required.

**Electronic supplementary material:**

The online version of this article (10.1007/s10555-020-09844-3) contains supplementary material, which is available to authorized users.

## Background

Were we to ask ourselves what we know about ovarian teratomas in children, what would the answer be? These lesions are the most common type of ovarian tumors in children. It is commonly accepted that they are germ cell–derived tumors, that they occur over a wide age range with the highest incidence in reproductive years, and that the majority occur as a purely teratomatous tumor. Nevertheless, some aspects of its pathology, classification, and management remain unclear [1, 2]. To name some of them: their embryology and genetic basis, their malignant potential, the possible use of ovarian-sparing operative techniques, and the suitability of chemotherapy in their treatment. Different staging systems, histopathologic classification, and risk stratification are important factors impeding the development of an appropriate treatment protocol. Additional factors include the lack of consistent terminology in the literature and analyzing pediatric and adult patients together [3]. Our knowledge of ovarian teratomas in children is far from complete, and much remains to be discovered.

An important measure of general health and social well-being is overall reproductive health, which is of great importance in the context of females with ovarian malignancies. As one of the most common ovarian neoplastic lesions requiring surgical treatment, ovarian teratoma in pediatric patients should be an important area of focus for clinicians, researchers, and policymaking groups due to the implications it poses for the well-being of children. An optimal consensus management strategy should be based on a detailed analysis of all possible factors, and a complete picture of the needs of individual patients [4]. Paucity of research dedicated exclusively to both mature and immature teratomas of the ovary in children contributes to decision-making difficulties.

To further advance the management of pediatric ovarian teratomas, additional research is needed to clarify this complex topic. Here, we provide a scoping review of the primary research related to the subject. To our knowledge, there is no published synthesis of the literature surrounding ovarian teratomas in children using scoping review methodology. Importantly, review studies can only be as good as their component parts [5, 6], and there exists a paucity of high-quality research on this topic.

## Methods

The scoping review followed the methodological framework developed by Arksey and O’Malley and incorporated additional scoping review recommendations made by Levac et al. [7, 8]. The protocol is available on request from the corresponding author. We followed the Preferred Reporting Items for Systematic reviews and Meta-Analyses extension for Scoping Reviews (PRISMA-ScR) Checklist (Additional File 1) [9]. Our review was conducted in five broad stages; each of which is outlined below. As mentioned above, this scoping review relates to primary research. An initial literature search of the topic revealed that there is a paucity of original primary studies dedicated exclusively to mature and immature teratomas of the ovary. To express the real current state of art upon the topic, we decided to include only this kind of studies in our review.

### Stage 1: Identifying the research questions

As we are becoming increasingly familiar with the literature, we have identified the following guiding questions in the area:

1. What are the key topics covered by selected studies?
2. What are the topics that are most amenable?
3. Are there certain areas of ovarian teratomas in children that can be explored more fully than others?
4. What are the key gaps in the existing knowledge?
5. Which areas need more research?
6. Are there reasons for certain areas being under-researched?

### Stages 2 and 3: Identifying and selecting relevant studies

The database search was run by one of the authors. Article selection and review took approximately 1 month and were completed by all the authors. Final terms were determined after an initial broad search using MEDLINE, which was used to identify MESH headings and alternative terms used in relevant papers. Using formerly described guidelines [10, 11], in consultation with a subject specialist librarian, we have developed a PubMed-specific search strategy. The following electronic databases were searched: (1) PubMed, (2) Web of Science, (3) CINAHL, (4) Cochrane Central Register of Controlled Trials. We also conducted a thorough scan of relevant gray literature (OpenGrey and Google). We limited our search to those with English language abstracts published between 1999 and 2019.

**Fig. 1:**
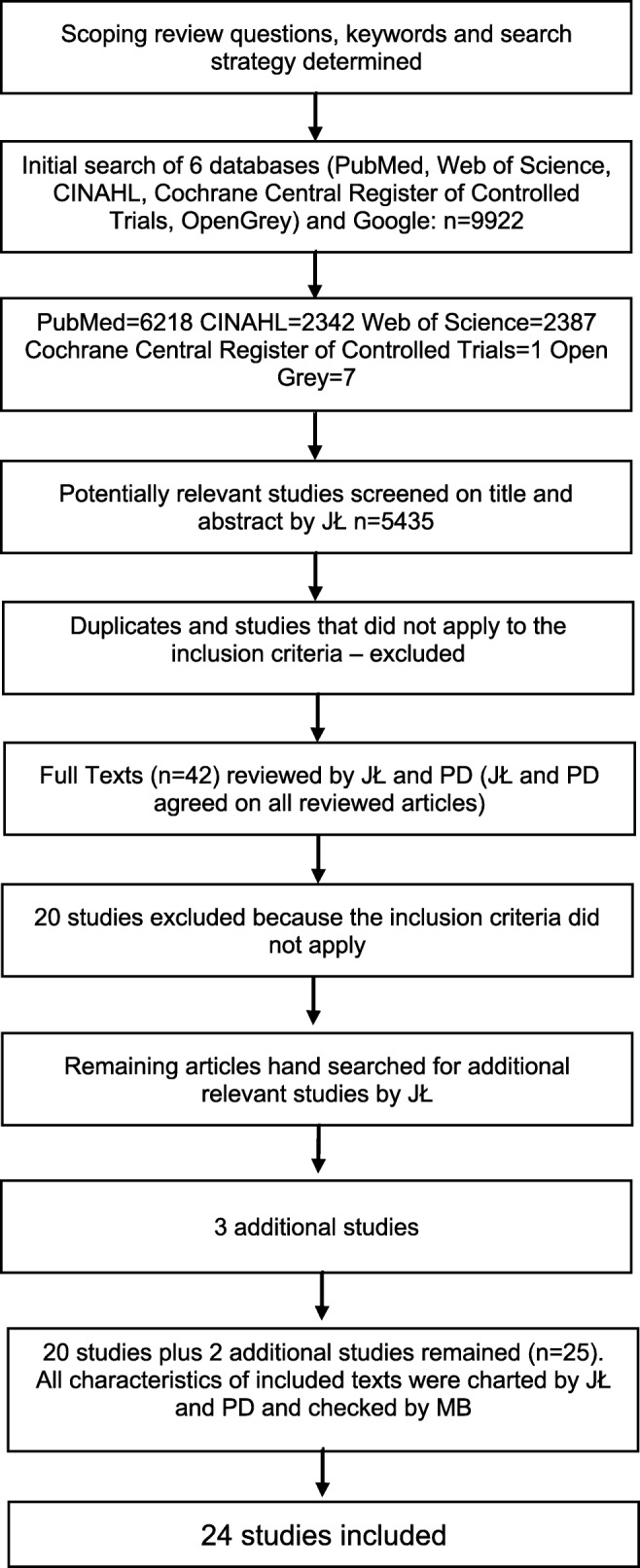
Selection process and search flow

The inclusion criteria and search strategy are shown in Table 1. The review team started the process by reviewing together a small sample of studies in order to ensure that there was an agreed common understanding about the inclusion and exclusion criteria. Disagreements about the papers were discussed both midway and at the end of the process. The selection process and search flow are shown in Fig. 1.

**Table 1:** Inclusion criteria and search strategy

Inclusion criteria	• Written in English
	• Reports primary research
	• Concerns ovarian teratoma in pediatric age (0–18 years)
	• Does not concern lesion other than teratoma (e.g., all germ cell tumors)
	• Study date 1999–2019
Keywords considered	Ovarian teratoma: Teratoma, Ovarian; Ovarian Neoplasms; Dermoid Cyst, Ovarian; Ovary Neoplasms; Neoplasms, Ovary
	Teratoma: Dysembryoma; Teratoid Tumor; Teratoma, Benign; Teratoma, Cystic; Teratoma, Immature; Teratoma, Malignant; Teratoma, Mature; Benign Neoplasms; Malignancy; Malignant Neoplasms; Neoplasia; Neoplasm; Neoplasms, Benign; Tumors
	Ovary: Ovaries; Gonads
	Child: Adolescent; Child, Preschool; Infant; Children; Minors
Search strategy in PubMed	1. ((“Ovarian Neoplasms”[Mesh]) OR (“Teratoma”[Mesh]) AND (“Child”[Mesh]) OR (“ovarian teratoma in children” OR “ovarian teratoma in a child” OR “teratoma of the ovary in a child” OR “ovarian teratoma in children” OR “teratoma of the ovary in children” OR “pediatric ovarian teratoma”) NOT medline[sb])
	2. ((“Ovarian Neoplasms”[Mesh]) OR (“Teratoma”[Mesh]) AND (“Child”[Mesh]) OR (teratoma* AND child* NOT medline[sb]))
Search strategy in Web of Science	1. # 1 (ALL = (teratoma AND child)) AND LANGUAGE: (English)
	2. # 2 (ALL = (teratoma AND ovary)) AND
	LANGUAGE: (English) 3. (#1 OR #2) AND LANGUAGE: (English)

### Stage 4: Charting the data

The data reported in the eligible papers were charted in an Excel spreadsheet. Characteristics included publication details, authors, year of publication, study location, study type (e.g., retrospective study), study group, aims of the study, overview of methods, outcome measures, and results. The categories for this spreadsheet were informed largely by Armstrong et al. [12].

### Stage 5: Collating, summarizing, and reporting the results

An overview of all material. We let the content of the included studies guide our theme development and identified and highlighted certain patterns across the papers in our study in the charting exercise. Three distinct steps were conducted: (1) analysis including descriptive numerical summary analysis and thematic analysis; (2) reporting the results and producing the outcome that refers to the overall purpose or research question; (3) discussing implications for future research and practice. We grouped the studies by the type of settings along with the measures used and broad findings. The remainder of this review will present the main points of research within the research questions described above. The review will conclude outlining the knowledge gaps that exist in addressing the primary question.

## Results

The search flow is demonstrated in Fig. 1. We identified 24 studies from 11 countries. Eighteen studies were retrospective, 3 were prospective, and 3 were experimental. There were 6 studies concerning mature teratomas, 5 regarded immature teratomas, and 13 included both types of the tumor. Overall, 9 of the studies concerned more than 50 patients. The journal’s title, lead author, place of origin, year of publication, title, study type, population, age group, study group, aims, overview of the methods, outcome measures, and main results related to each study are presented in Supplemental Fig. 1. We also included future study questions indicated by the authors.

Our charting exercise revealed 7 major branches of research within the topic of ovarian teratoma in pediatric population: recurrence rate/relapse and follow-up strategy, malignant potential, prognostic factors, use of sparing surgery, differences between the use of laparoscopy and laparotomy, use of chemotherapy, and additional examinations to test the character of the lesion (immature *vs*. mature). These issues were most commonly indicated as the main focus of corresponding studies. Additional topics less frequently mentioned or not indicated as the main aim of the studies were as follows: incidence of familiar forms, possible use of pharmacotherapy, use of contralateral biopsy, use and differences in staging procedures, alpha-fetoprotein (AFP) cutoff level, imaging features, need for central pathology review, importance of spillage, grading, gliomatosis peritonei, associated defects, and discovery of new diagnostic factors. Table 2 presents an overview of the study topics. A detailed description of the main topics is presented below. [13–36].

**Table 2:** Overview of the study topics

TOPIC (main with red color)																							
Article No.	Recurrence rate/relapse and follow up strategy	differences between LS and LT	Prognostic factors	Incidence of familiar forms	Possible use of pharmacotherapy	Use of sparing surgery	Malignant potential	Use of contralateral biopsy	Grading	Use and differences in staging procedures	Alpha-fetoprotein (AFP) cutoff level	Imaging features	Use of chemotherapy	Need for central pathology review	Importance of spillage	Gliomatosis peritonei	Associated defects	Additional examinations to test the immature or mature caracter	Discovery of new diagnostic factors - e.g. genotyping	Overall topics included	Mature	Immature	Both
1	+	+																		2	+		
2	+	+						+												3	+		
3	+	+	+	+	+	+								+	+	+				9	+	+	
4	+		+				+	+												4	+	+	
5	+		+			+			+		+	+								6		+	
6	+	+				+			+			+			+					6	+	+	
7	+						+		+		+		+	+		+				7		+	
8	+		+			+										+				4	+	+	
9																	+			+		+	
10	+						+		+				+	+						5	+	+	
11	+		+																+	3		+	
12	+						+						+	+	+					5	+	+	
13	+		+				+				+		+							4	+	+	
14												+		+				+		3	+	+	
15	+					+		+						+						4	+		
16																		+		+	+	+	
17		+				+									+					3	+		
18	+			+			+								+					4	+	+	
19												+								1	+	+	
20		+													+					2	+		
21	+		+				+		+	+			+							6		+	
22	+					+			+											3	+	+	
23	+								+				+			+				4	+	+	
24																			+		+		
Overall number of the studies	14	6	7	2	1	8	7	3	7	1	3	4	6	6	6	4	1	3	2		17	16	13
Number of the studies including more than 50 cases	7	4	3	1	1	3	3	1	2	1	1	0	3	2	1	2	1	0	0		3	2	4
Number of the studies concerning mature teratoma	3	0	0	0	0	3	0	2	1	0	0	0	0	1	2	0	0	0	1				
Number of the studies concerning immature teratoma	3	4	3	0	0	1	2	0	3	1	2	1	2	1	0	1	1	1	1				
Number of the studies concerning both types of the tumor	8	2	4	2	1	3	5	1	3	0	1	3	4	4	4	3	0	1	0				

+ indicates that the topic from the column appeared in the specific article(article numbered 1 to 24 as indicated in the Supplemental Fig. 1)

### Recurrence rate/relapse and follow-up strategy

Four studies indicated recurrence rate/relapse and follow-up strategy as a main topic, and it was mentioned in 13 others. Three of these studies concerned mature teratoma and 4 concerned immature teratomas. In all of these studies, a recurrence rate was provided. Seven studies concerned more than 50 patients. There was no uniform follow-up strategy; nevertheless, only 5 studies mentioned it as a limitation. Four publications indicated the need for prolonged follow-up due to the risk of contralateral ovarian teratoma [13–29].

### Malignant potential

Four studies indicated malignant potential as a main topic, and it was mentioned in 3 others. Two studies concerned immature teratoma and 5 regarded both mature and immature teratomas. Three papers concerned more than 50 patients. In two studies, a patient with malignant histology was characterized as having an immature teratoma; however, the full pathology report was not included. In a study by Cushing et al., a malignant relapse was described in one patient with immature teratoma and a highly elevated AFP level. In a German study, there were no malignant relapses in the group that received chemotherapy. Biskup et al. described teratomas with malignant transformation in 6 out of 246 cases. One study, which excluded patients with highly elevated AFP levels (above 1000 ng/mL), revealed that the grade was the most important risk factor for relapse in ovarian immature teratoma and adjuvant chemotherapy did not decreased relapses. However, in this study, no detailed pathology report of the relapses was provided [16, 19, 22, 24, 25, 27, 28].

### Prognostic factors

Three studies indicated prognostic factors as a main topic, and it was mentioned in 4 others. Three papers concerned immature teratoma and 4 included both types. In all of these studies, a recurrence rate was provided. Three studies concerned more than 50 patients. The studies that succeed in the indication of prognostic factors revealed that older age and higher AFP level are associated with higher grade of immaturity, relapses occur in patients with overexpression of p53, and incomplete resection or grading is a possible risk factor of relapse. Nevertheless, all of these studies highlighted the need for further research in these fields [15–17, 23, 25, 28].

### Differences between the use of laparoscopy and laparotomy

Two studies indicated differences between the use of laparoscopy and laparotomy as a main topic, and it was mentioned in 4 others. Four studies concerned immature teratoma and 2 included both types. Also, four papers concerned more than 50 patients. One of the studies did not find a significant association between the type of surgery utilized and dermoid recurrence. Another one revealed that patients managed laparoscopically had shorter hospital stay. In studies by Savasi et al. and Childress et al., a significantly higher rate of cyst rupture was experienced during laparoscopic cystectomy compared with excision *via* laparotomy. These studies also found that the length of hospital stay was significantly shorter in the laparoscopy group compared with that in the laparotomy group. As these studies lacked uniform surgical guidelines for the use of laparoscopy in ovarian teratoma and mentioned that varying surgical techniques were used for cyst dissection, an objective comparison is very difficult [13–15, 18, 31, 32].

### Use of sparing surgery

Two studies indicated the use of sparing surgery as a main topic, and it was mentioned in 6 others. One paper concerned immature teratoma, 3 concerned mature teratoma, and 3 included both types. Three manuscripts concerned more than 50 patients. In all except two studies (regarding exclusively mature teratoma) oophorectomy predominated. Three studies highlighted the need for creating guidelines for the use of ovarian-sparing surgery in ovarian teratomas [15, 17, 18, 20, 26, 29, 31].

### Use of chemotherapy

Two studies indicated the use of chemotherapy as a main topic, and it was mentioned in 4 others. Two papers concerned immature teratoma and 4 both types. Half of the manuscripts concerned more than 50 patients. In a previously cited study by Cushing et al., in a group of patients with immature teratoma without postoperative chemotherapy, there was only one malignant relapse in a child with highly elevated AFP level preoperatively. A study by Göbel et al. revealed that relapse rate in patients with mature and immature teratoma decreased significantly only after complete resection. Incomplete resection was also indicated by Lo Curto et al. as a possible risk factor in their study where the patients with grades 2 and 3 tumors received chemotherapy and relapsed in 4 cases of incomplete resection. Grade was the most important factor of relapse in a study by Pashankar et al. and adjuvant chemotherapy did not decrease relapses in the pediatric cohort. The need for chemotherapy was confirmed in the case of malignant relapse or malignant transformation in all of the papers. None of the studies were able to assess the real efficiency of chemotherapy mostly due to lack of uniform diagnostic and treatment methods across the studies [19, 22, 25, 28, 30].

### Additional examinations to test the immature or mature character

Two studies indicated this issue as a main topic. One of the studies concerned both tumor types, and the other included mature teratoma. Neither of them concerned more than 50 patients. A high-uptake ratio of gallium-67 in benign teratoma was indicated in one manuscript. A study by Gu et al. revealed that GFAP (glial fibrillary acidic protein) is highly expressed in the nerve tissue of mature teratomas and is low in that of immature ones [33, 34].

## Discussion

This review allowed us to develop an overview of the available literature in the field. Our findings indicate a paucity of research focusing specifically on ovarian teratoma in pediatric population. This interesting group of neoplasms presents diverse biological behavior and continues to be the cause of many diagnostically and therapeutically challenging issues. Marks of the controversy regarding these tumors are also reflected in the nomenclature of these lesions across the studies. For instance, multiplicity of names describing immature teratomas (immature teratoma, malignant teratoma, teratoma with malignant elements, immature teratoma with malignant behavior) renders universally applicable classification of these lesions very difficult [20, 37–41].

Whereas the key topics found by our review concerned diagnosis and treatment, only a few studies concentrated on basic research investigating the real nature of these lesions. Moreover, most of the studies were retrospective and less than a half included more than 50 patients. An important limitation in exploring the issue is the low incidence of ovarian teratomas among all pediatric diseases. Older girls may be also referred to adult gynecological departments, thus escaping the pediatric surgical database. Therefore, answering two of the study questions—what are the topics that are most amenable and are there certain areas of ovarian teratomas that can be explored more fully than others?—it seems to be much easier and common to examine the diagnosis and treatment actually applied in the management of ovarian teratoma than defining its nature and behavior by means of prospective and experimental studies. Most of the studies left their study questions without clear answers, highlighting the need for further research. Therefore, we answered indirectly one of the other study questions: are there reasons for certain areas to be under-researched? Furthermore, almost all of the studies highlighted their limitations. Except the ones indicated above, we should not forget about the lack of uniform diagnostic, treatment, and follow-up methods across the studies. Referring to adult population and old studies is another important obstacle. For many years, the therapeutic principles elaborated for adult patients were applied by pediatric surgeons too. However, epidemiology and clinical nature of ovarian tumors in pediatric population differ to a large extent from those of women so such direct transfer of management approach seems nowadays unwarranted [42, 43]. Difficulties in studying ovarian teratomas in children seem to be present in all aspects beginning from their correct pathological classification as an example. Obtaining the correct pathology report is of crucial importance in this case. Performing a central pathomorphological examination can be helpful as it was mentioned by some of the studies [2, 15, 19, 20, 22, 24, 26, 33].

The use of scoping review methodology was particularly advantageous as we were not restricted to tight inclusion criteria. However, we were limited in a number of ways. We were probably unable to find all relevant studies. A quantitative synthesis may have revealed additional insights. Not encompassing all studies concerning ovarian teratomas in wider study groups (e.g., all age groups, all ovarian teratomas, or all germ cell tumors included) hindered our ability to fully analyze the topic in the context of less thematic restricted studies.

In conclusion, this scoping review has revealed a number of knowledge gaps in the evidence base around ovarian teratoma in pediatric age. Overall, this topic has not been extensively explored, and more research dedicated exclusively to children is required. There is no doubt that contemporary medicine should not be a matter of chance. Evidence-based medicine is the key to good medical practice and making informed clinical decisions. The review revealed insufficient number of basic research and prospectively designed high-quality multi-institutional studies. Additionally, further research is necessary to improve understanding of the biology, genetics, and prognostic factors of ovarian teratomas in pediatric population to establish the optimal management and develop novel therapeutic approaches. More studies are needed to identify the potential harms of current treatment methods and to evaluate their effectiveness.

## Electronic supplementary material

ESM 1 (PNG 5138 kb)

High Resolution Image (TIFF 47936 kb)

## Data Availability

The datasets used and analyzed during the current study are available from the corresponding author on reasonable request.
